# Piceatannol, a Structural Analog of Resveratrol, Is an Apoptosis Inducer and a Multidrug Resistance Modulator in HL-60 Human Acute Myeloid Leukemia Cells

**DOI:** 10.3390/ijms221910597

**Published:** 2021-09-30

**Authors:** Kamila Siedlecka-Kroplewska, Agata Wrońska, Zbigniew Kmieć

**Affiliations:** Department of Histology, Medical University of Gdańsk, 80-211 Gdańsk, Poland; awronska@gumed.edu.pl (A.W.); zkmiec@gumed.edu.pl (Z.K.)

**Keywords:** acute myeloid leukemia, HL-60 cells, piceatannol, apoptosis, multidrug resistance, P-glycoprotein, MRP1, BCRP

## Abstract

Acute myeloid leukemia is characterized by uncontrolled clonal proliferation of abnormal myeloid progenitor cells. Despite recent advances in the treatment of this disease, the prognosis and overall long-term survival for patients remain poor, which drives the search for new chemotherapeutics and treatment strategies. Piceatannol, a polyphenolic compound present in grapes and wine, appears to be a promising chemotherapeutic agent in the treatment of leukemia. The aim of the present study was to examine whether piceatannol induces autophagy and/or apoptosis in HL-60 human acute myeloid leukemia cells and whether HL-60 cells are able to acquire resistance to piceatannol toxicity. We found that piceatannol at the IC_90_ concentration of 14 µM did not induce autophagy in HL-60 cells. However, it induced caspase-dependent apoptosis characterized by phosphatidylserine externalization, disruption of the mitochondrial membrane potential, caspase-3 activation, internucleosomal DNA fragmentation, PARP1 cleavage, chromatin condensation, and fragmentation of cell nuclei. Our findings also imply that HL-60 cells are able to acquire resistance to piceatannol toxicity via mechanisms related to MRP1 activity. Our results suggest that the use of piceatannol as a potential chemotherapeutic agent may be associated with the risk of multidrug resistance, warranting its use in combination with other chemotherapeutic agents.

## 1. Introduction

Acute myeloid leukemia (AML) is characterized by uncontrolled clonal proliferation of abnormal myeloid progenitor cells that accumulate mainly in the bone marrow and blood [1]. AML is the second most common type of leukemia in children and the most common acute leukemia in adults [2,3]. Despite recent advances in the treatment strategies of AML, the prognosis and overall long-term survival for patients, especially for those over 65 years old, remain poor [3,4]. The etiology of AML is related to genetic mutations that may appear de novo or as a consequence of prior cytotoxic chemotherapy or radiation [1,3]. AML is a highly heterogenous disease characterized by the presence of heterogeneous subpopulations of cells with distinct genetic and epigenetic alterations [5]. Treatment strategies of AML are based primarily on chemotherapy and allogeneic stem cell transplantation for eligible patients [3,6,7]. The backbone of initial induction chemotherapy has not changed for several decades and is based mainly on a combination of cytarabine and anthracyclines [6,7]. Another important therapeutic strategy recently introduced for AML treatment is based on the use of hypomethylating agents (HMA) such as decitabine [8]. The major problems of chemotherapy of AML are treatment-related toxicity and mortality, cellular heterogeneity, as well as the occurrence of multidrug resistance (MDR) [5,6,7,8,9,10]. MDR results from the overexpression of transport proteins belonging to the ATP-binding cassette (ABC) superfamily [11,12,13,14]. ABC transporters are transmembrane proteins present in normal tissues as well as in neoplasms [15,16,17,18,19,20,21,22,23,24,25,26,27]. They function in the transport of metabolites, hormones, ions, toxic compounds and other substrates 12,21,26]. Many ABC efflux pumps have been demonstrated to facilitate removal of anticancer drugs from cancer cells [11,13]. The overexpression of ABC transporters in cancer cells may lead to increased drug efflux resulting in resistance to chemotherapy [13,14]. There are many well-characterized ABC efflux pumps including P-glycoprotein (P-gp), multidrug resistance-associated protein 1 (MRP1), and breast cancer resistance protein (BCRP) [12,20,21,22,23,24,25,26,27]. Accumulating evidence suggests that P-gp, MRP1 and BCRP expression may be involved in multidrug resistance in AML [9,10,28,29,30,31,32,33].

**Figure 1:**
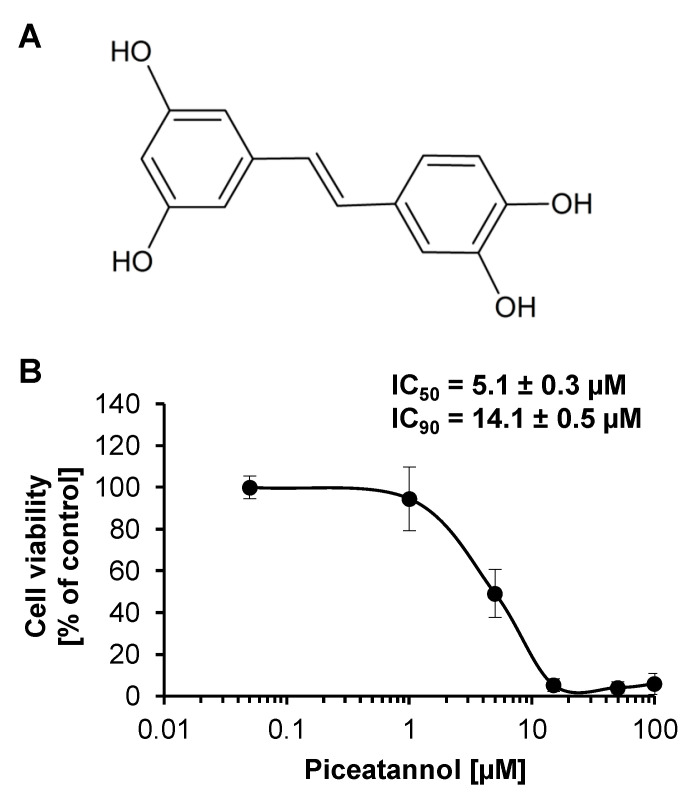
Effect of piceatannol on the viability of HL-60 cells. (**A**) The chemical structure of piceatannol. (**B**) Neutral red uptake assay. HL-60 cells were incubated in the absence (control) or presence of piceatannol for 72 h. Data are presented as means ± SD of three independent experiments in duplicates.

Polyphenolic compounds have recently attracted the attention of researchers due to their pleiotropic biological activities. Piceatannol (*trans*-3,3′,4′,5-tetrahydroxystilbene, Figure 1A) is a naturally occurring polyphenol and a structural analog of resveratrol (*trans*-3,4′,5-trihydroxystilbene) found in grapes, berries, passion fruit, and wine [34,35,36,37]. It has been demonstrated to exhibit a wide range of biological properties including anti-oxidative, antimicrobial, anti-inflammatory, antidiabetic, estrogenic, neuroprotective, and cardioprotective activities [38,39,40,41,42,43,44]. Piceatannol (Figure 1A) has been reported to inhibit growth and induce apoptosis in many human cancer cell lines including leukemia, lymphoma, and melanoma as well as breast, prostate, bladder, and colon cancer [45,46,47,48,49,50,51,52,53,54,55,56,57,58]. Moreover, piceatannol has been found to induce autophagy in different cancer cell lines such as U2OS human osteosarcoma and MOLT-4 human leukemia cells [59,60]. Resveratrol, which is structurally similar to piceatannol, has recently been demonstrated to reverse multidrug resistance in several cancer cell lines including adriamycin-resistant HL-60/ADR cell line [61,62,63]. However, in our previous study, we have reported that MOLT-4 cells are able to acquire resistance to piceatannol toxicity after prolonged exposure to this polyphenol [59].

The aim of the present study was to examine the effects of piceatannol on HL-60 human acute myeloid leukemia cells regarding its ability to induce autophagy and/or apoptosis in HL-60 cells as well as the ability of HL-60 cells to acquire resistance to piceatannol toxicity. The HL60 human acute promyelocytic leukemia cell line is a widely used experimental model for studying mechanisms of action of chemotherapeutic drugs as well as potential new chemotherapeutic agents [64,65]. We found that piceatannol at the IC_90_ concentration of 14 µM did not lead to cell cycle arrest or autophagy; however, it induced caspase-dependent apoptosis in HL-60 cells. Moreover, to our knowledge, this is the first study demonstrating that HL-60 cells are able to acquire resistance to piceatannol toxicity after prolonged exposure to this polyphenol.

## 2. Results

### 2.1. Effect of Piceatannol on the Viability and Cell Cycle Distribution of HL-60 Cells

Since the calculation of IC_50_ and IC_90_ values is recommended to evaluate the cytotoxic potency of studied compounds [66], we calculated these values for piceatannol. The concentration of piceatannol required to inhibit growth of HL-60 cells by 50% (IC_50_) and 90% (IC_90_), estimated after 72 h of treatment of cells with this compound, was 5.1 µM and 14.1 µM, respectively (Figure 1B). Further experiments were performed at piceatannol concentration of 14 µM, corresponding to the IC_90_ value.

**Figure 2:**
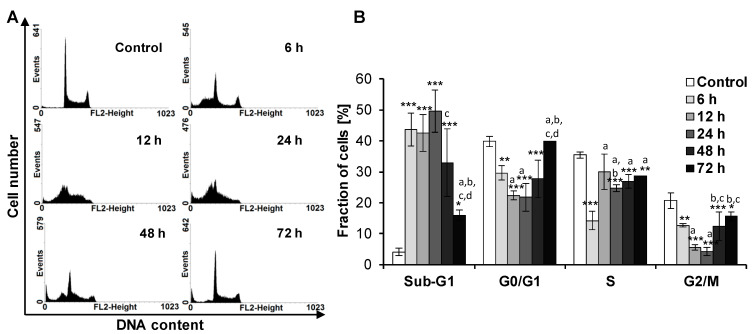
Effect of piceatannol on the cell cycle distribution of HL-60 cells. Cells were incubated with piceatannol (IC_90_ = 14 µM) for 6, 12, 24, 48, and 72 h (PI staining). (**A**) Time-course changes in the cell cycle distribution of piceatannol-treated HL-60 cells. Data correspond to one representative experiment. Similar results were obtained in a total of three independent experiments in duplicates. (**B**) Percentage of cells of the sub-G1 fraction and phases of the cell cycle. Data are presented as means ± SD of three independent experiments in duplicates. * *p* < 0.05, ** *p* < 0.01, *** *p* < 0.001, statistically significant differences compared to control (untreated cells); ^a^ *p* < 0.05, statistically significant differences compared to cells treated with piceatannol for 6 h; ^b^ *p* < 0.05, compared to cells treated with piceatannol for 12 h; ^c^ *p* < 0.05, compared to cells treated with piceatannol for 24 h; ^d^ *p* < 0.05, compared to cells treated with piceatannol for 48 h (One-way ANOVA, Tukey’s post hoc analysis).

We found that treatment of HL-60 cells with piceatannol at the IC_90_ concentration was not accompanied by the cell cycle arrest (Figure 2A,B). Compared to control (untreated cells), incubation with piceatannol for 6–72 h led to a statistically significant increase in the number of cells in the sub-G1 fraction, suggesting apoptotic DNA degradation (Figure 2B). After 48 h, the percentage of cells in the sub-G1 fraction decreased in comparison to cells treated with piceatannol for a shorter time, i.e., 6, 12, or 24 h. However, these changes were statistically significant only between 48 h and 24 h drug exposure. Moreover, following 72 h of incubation of HL-60 cells with piceatannol a statistically significant decrease in the sub-G1 fraction was observed compared to 6, 12, 24, and 48 h of exposure.

### 2.2. Effect of Piceatannol on Reactive Oxygen Species Production in HL-60 Cells

**Figure 3:**
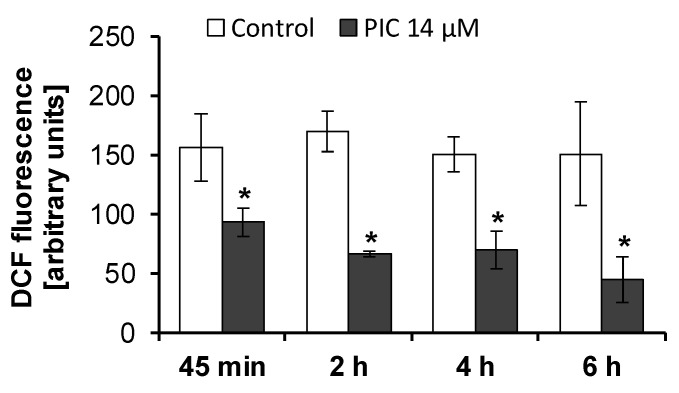
Effect of piceatannol on ROS production in HL-60 cells. Cells were incubated with piceatannol (IC_90_ = 14 µM) for 45 min and 2, 4, or 6 h. Data are presented as means ± SD of three independent experiments in duplicates. * *p* < 0.05, statistically significant differences compared to control (Mann–Whitney U test).

In order to find out whether piceatannol is able to induce oxidative stress in HL-60 cells, we examined its effect on the intracellular production of reactive oxygen species (ROS). Compared to control, the formation of ROS in HL-60 cells decreased after treatment with piceatannol (IC_90_). After 2 and 4 h of incubation with this compound the intracellular ROS production decreased more than two-fold, whereas after 6 h of treatment it decreased three-fold in comparison to control (Figure 3).

### 2.3. Effect of Piceatannol on Autophagy Pathways

**Figure 4:**
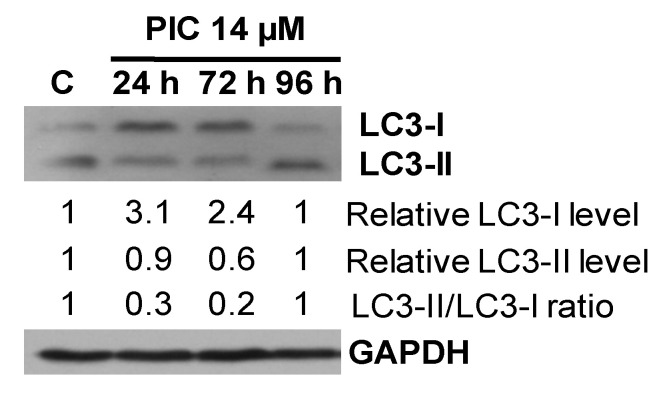
Effect of piceatannol on autophagy pathways in HL-60 cells. Cells were treated with piceatannol (PIC, IC_90_ = 14 µM) for 24, 72, and 96 h. The relative levels of LC3-I and LC3-II proteins normalized to loading control GAPDH were quantitated by densitometry. C—control (untreated cells), PIC—piceatannol. Similar results were obtained in three independent experiments.

The conversion of LC3-I to LC3-II protein belongs to characteristics of autophagy and indicates the formation of autophagic vacuoles [67,68] We have previously shown that piceatannol induced autophagy in MOLT-4 human leukemia cells [59]. Therefore, we decided to examine whether piceatannol is able to modulate autophagy pathways in HL-60 human leukemia cells. The Western blotting analysis revealed that the relative LC3-I level (normalized to loading control GAPDH) in HL-60 cells increased after 24 and 72 h of treatment with piceatannol (IC_90_) by a factor of 3.4 and 2.1, respectively, compared to control (Figure 4). Moreover, after 24 and 72 h of exposure of cells to piceatannol the relative LC3-II level (normalized to loading control GAPDH) decreased compared to control and was 0.9 and 0.6, respectively (Figure 4). Noteworthy, after 96 h of exposure both relative LC3-II and LC3-I protein levels did not change in comparison to control. The calculated LC3-II/LC3-I ratio indicates that there was no conversion of LC3-I to LC3-II. Following 24, 72, and 96 h of incubation with piceatannol, the LC3-II/LC3-I ratios were 0.3, 0.2, and 1.0, respectively.

**Figure 5:**
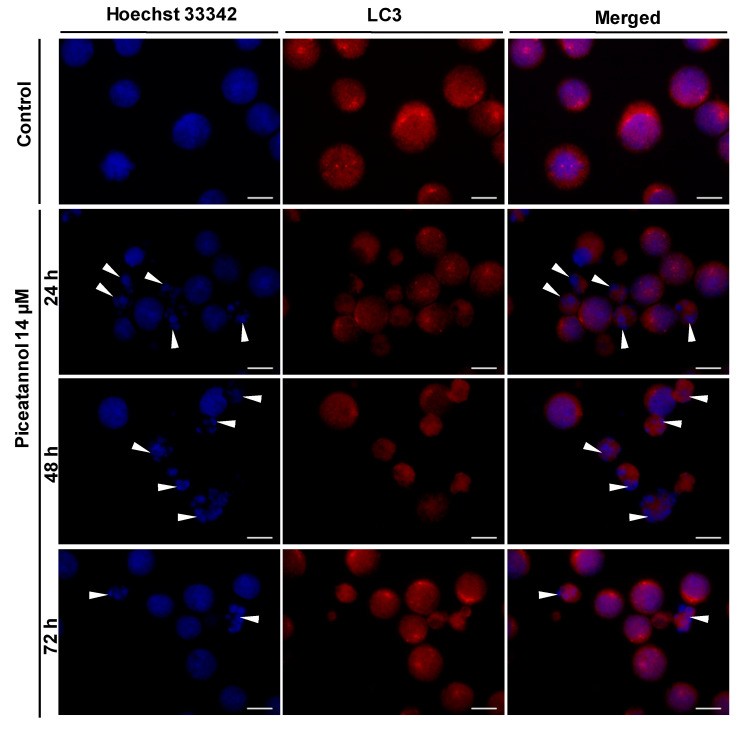
Detection of LC3 protein in HL-60 cells. Cells were treated with piceatannol (IC_90_ = 14 µM) for 24, 48, and 72 h. Cells were incubated with primary anti-LC3 antibody and subsequently with Cy3-conjugated secondary antibody. Following staining with Hoechst 33342, cells were examined by fluorescence microscopy. Data are representative of three independent experiments. Bars, 10 µm; control—untreated cells; arrowheads–cells with fragmented nuclei.

In agreement with the results of the Western blotting analysis, fluorescence micrographs did not show accumulation of autophagic vacuoles in piceatannol-treated HL60 cells (Figure 5). In both control and piceatannol-treated cells, LC3 staining was mostly diffuse, suggesting cytosolic localization of the LC3 protein (Figure 5).

### 2.4. Induction of Cell Death in Piceatannol-Treated HL-60 Cells

**Figure 6:**
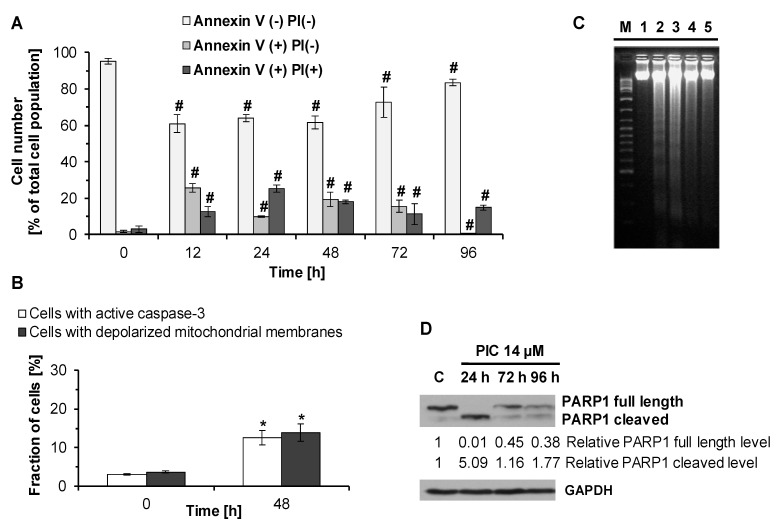
Detection of apoptosis in piceatannol-treated HL-60 cells. (**A**) Effect of piceatannol on phosphatidylserine externalization and loss of plasma membrane integrity in HL-60 cells. Cells were treated with piceatannol (IC_90_ = 14 µM) for 12, 24, 48, 72, and 96 h. Data are presented as means ± SD of three independent experiments. ^#^ *p* < 0.0001, statistically significant differences compared to control (One-way ANOVA, Dunnett’s post hoc analysis). (**B**) Effect of piceatannol on caspase-3 activation and changes of the mitochondrial membrane potential in HL-60 cells. Cells were treated with piceatannol (IC_90_ = 14 µM) for 48 h. Data are presented as means ± SD of three independent experiments. * *p* < 0.05, statistically significant differences compared to control—untreated cells (Mann–Whitney U test). (**C**) Effect of piceatannol on DNA fragmentation in HL-60 cells. Cells were exposed to piceatannol (IC_90_ = 14 µM) for 12, 24, 48, and 72 h. M, 100–10,000 bp DNA marker; lane 1, control (untreated cells); lanes 2–5, 12, 24, 48, and 72 h of piceatannol treatment, respectively. Data are representative of three independent experiments. (**D**) Effect of piceatannol on PARP1 cleavage in HL-60 cells. Cells were exposed to piceatannol (PIC, IC_90_ = 14 µM) for 24, 72, and 96 h. C–control (untreated cells). The relative levels of full-length PARP1 (113 kDa) and cleaved PARP1 (89 kDa) normalized to loading control GAPDH were quantitated by densitometry. Similar results were obtained in three independent experiments.

In order to examine the mechanism of cell death induced by piceatannol in HL-60 cells, we studied the compound’s effects on phosphatidylserine externalization, caspase-3 activation, changes in mitochondrial membrane potential, internucleosomal DNA fragmentation, cleavage of poly(ADP-ribose) polymerase (PARP-1), chromatin condensation and fragmentation of cell nuclei. Our results revealed that treatment of HL-60 cells with piceatannol (IC_90_) led to phosphatidylserine externalization in HL-60 cells (Figure 6A). Compared to control (untreated cells), after 12, 24, 48, and 72 h of incubation with the compound, an increase was observed in fractions of early apoptotic cells (annexin V^+^/PI^−^) and late apoptotic/necrotic cells (annexin V^+^/PI^+^), accompanied by a corresponding decrease in the fraction of viable cells (annexin V^−^/PI^−^). However, after 96 h of incubation with piceatannol, these changes were less prominent than after shorter periods of exposure. Of note, at this time point, early apoptotic cells constituted only 0.8 % (±0.1) of the total cell population (Figure 6A).

Our results also revealed that piceatannol-induced death of HL-60 cells was associated with caspase-3 activation and loss of the mitochondrial membrane potential (Figure 6B). After 48 h of treatment of cells with piceatannol (IC_90_), cells with active caspase-3 constituted 13% of the total cell population, whereas the fraction of cells with reduced mitochondrial membrane potential was 14% (Figure 6B).

As shown in Figure 6C, piceatannol (IC_90_) led to internucleosomal DNA fragmentation in HL-60 cells. This effect was more prominent following 12 and 24 h than after 48 and 72 h of exposure of cells to piceatannol. In line with the above findings, the Western blotting analysis showed PARP1 cleavage in piceatannol-treated HL-60 cells (Figure 6D). Noteworthy, this effect was more prominent after 24 than 72 or 96 h of treatment with piceatannol (IC_90_). Following exposure of HL-60 cells to piceatannol for 24, 72, and 96 h, the levels of PARP1 cleaved (89 kDa) relative to control were 5.09, 1.16, and 1.77, respectively (Figure 6D). The relative levels of PARP1 full length after 24, 72, and 96 h were 0.01, 0.45, and 0.38, respectively (Figure 6D).

Piceatannol-treated HL60 cells also exhibited other apoptotic characteristics. Fluorescence micrographs showed the presence of condensed and fragmented nuclei in cells exposed to piceatannol (IC_90_) (Figure 5). These morphological changes were more prominent after 24 h and 48 h than 72 h of drug treatment (Figure 5).

### 2.5. Effects of Piceatannol on the Activity and Expression of P-gp

**Figure 7:**
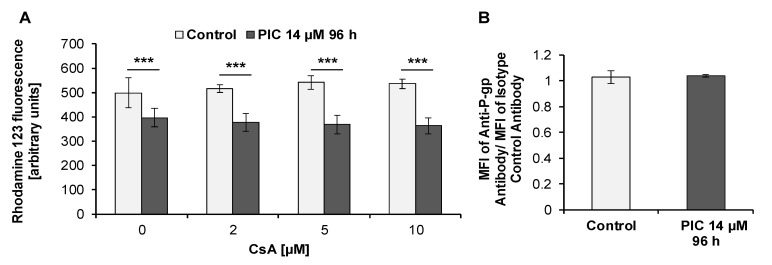
Detection of P-gp in HL-60 cells. Cells were incubated with piceatannol (PIC, IC_90_ = 14 µM) for 96 h. (**A**) P-gp activity analysis (flow cytometry analysis). Rhodamine 123 uptake/retention assay was performed in the presence and absence of cyclosporin A (CsA). Data are presented as means ± SD of three independent experiments in duplicates. *** *p* < 0.001, statistically significant differences between piceatannol-treated cells and untreated cells incubated in the absence or presence of CsA (One-way ANOVA, Tukey’s post hoc analysis). (**B**) Expression of P-gp in HL-60 cells. Control (untreated cells) and piceatannol-treated cells were stained with anti-P-gp (clone UIC2) antibody or isotype control antibody (flow cytometry analysis). Data are presented as means ± SD of three independent experiments in duplicates.

We have previously found that MOLT-4 leukemia cells acquire resistance to piceatannol toxicity, which may result from the induction of ABC transporters [59]. Some experiments such as the cell cycle analysis, examination of morphological changes, and phosphatidylserine externalization in HL-60 cells treated with piceatannol (IC_90_) revealed that toxic effects exerted by this compound diminished after longer treatment periods. Therefore, we decided to examine whether ABC transporters-mediated drug efflux mechanisms may be responsible for these effects. Our studies focused on the detection of three important ABC efflux pumps: P-gp, MRP1, and BCRP. To detect the activity of ABC transporters we performed the rhodamine 123 (Rho 123) uptake/retention assay, which is recommended for this purpose [69,70]. We also examined the expression of P-gp, MRP1, and BCRP at the protein level by flow cytometry. The flow cytometric analysis is considered a sensitive and useful method for the assessment of ABC transporters’ expression and function in hematological malignancies [70]. Of note, rhodamine 123 can be transported by P-gp and proteins belonging to the MRP family [71,72,73]. We studied the activity of selected ABC transporters after 96 h of incubation of HL-60 cells with piceatannol (IC_90_). The measured signals were then detectable by the experimental methods we used. We used the same experimental design in our previous study concerning similar effects of piceatannol in MOLT-4 cells [59]. In that study, we demonstrated that MOLT-4 cells are able to acquire resistance to piceatannol toxicity and P-gp activity may be responsible for these effects [59]. We have therefore decided to examine the activity of P-gp in HL-60 cells after 96 h treatment with piceatannol (IC_90_). We evaluated Rho 123 retention in the absence and presence of cyclosporin A (CsA), an inhibitor of P-gp [74]. Our results revealed that the mean fluorescence intensity of Rho 123, corresponding to its intracellular retention, decreased in piceatannol-treated cells in comparison to control (untreated) cells (Figure 7A). Both in the absence and in the presence of 2, 5, or 10 µM CsA, statistically significant differences were observed between piceatannol-treated and control cells. CsA did not inhibit Rho 123 efflux in control (untreated) cells or in piceatannol-treated cells (Figure 7A).

In agreement with the above findings, staining of HL-60 cells with the specific monoclonal anti-P-gp antibody (clone UIC2) did not reveal cell surface expression of P-gp (Figure 7B). We used the UIC2 clone which is one of the most commonly used antibodies for the assessment of P-gp expression [69,70]. There were not statistically significant differences between control (untreated) cells and piceatannol-treated cells. In control cells, the MFI shift was 1.03 (±0.05), whereas after treatment of cells with piceatannol (IC_90_) for 96 h the MFI shift was 1.04 (±0.01).

### 2.6. Effects of Piceatannol on the Activity and Expression of MRP1

**Figure 8:**
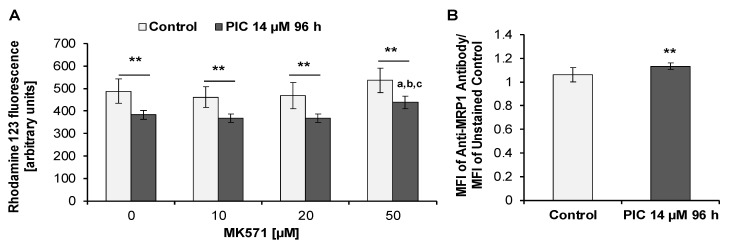
Detection of MRP1 in HL-60 cells. Cells were incubated with piceatannol (PIC, IC_90_ = 14 µM) for 96 h. (**A)** MRP1 activity analysis (flow cytometry analysis). Rhodamine 123 uptake/retention assay was performed in the presence and absence of MK571. Data are presented as means ± SD of three independent experiments in duplicates. ** *p* < 0.01, statistically significant differences between piceatannol-treated cells and untreated cells, both incubated in the absence or presence of MK571. ^a^ *p* < 0.001, statistically significant differences compared to piceatannol-treated cells incubated without MK571; ^b^ *p* < 0.001, statistically significant differences compared to piceatannol-treated cells incubated with 10 µM MK571; ^c^ *p* < 0.001, statistically significant differences compared to piceatannol-treated cells incubated with 20 µM MK571 (One-way ANOVA, Tukey’s post hoc analysis). (**B**) Expression of MRP1 in HL-60 cells. Control (untreated cells) and piceatannol-treated cells were stained with anti-MRP1 (QCRL-3) antibody (flow cytometry analysis). Data are presented as means ± SD of three independent experiments in duplicates. (Student’s *t*-test).

In order to detect the activity of MRP proteins, we examined Rho 123 retention in the presence and absence of MK571, a specific MRP inhibitor [75]. We found that after treatment of cells for 96 h with piceatannol (IC_90_) the intracellular Rho 123 level in the absence or presence of MK571 was significantly lower than in untreated (control) cells (Figure 8A). Moreover, there were no statistically significant changes between control cells incubated in the presence of 10 µM, 20 µM, and 50 µM MK571 and control cells incubated in the absence of MK571. In piceatannol-treated cells, in the presence of 50 µM MK571 a statistically significant increase in the intracellular Rho 123 level was noted compared to piceatannol-treated cells incubated at lower concentrations of MK571 or in the absence of MK571 (Figure 8A). The above results suggest that MRP proteins may be involved in Rho 123 efflux in HL-60 cells.

In agreement with the above findings, staining of HL-60 cells with the specific monoclonal anti-MRP1 antibody (clone QCRL-3) revealed the expression of MRP1 in piceatannol-treated cells (Figure 8B). There were statistically significant differences between control cells and piceatannol-treated cells. In control cells, the MFI shift was 1.05 (±0.06), whereas after exposure of cells to piceatannol (IC_90_) for 96 h, the MFI shift was 1.13 (±0.03).

**Figure 9:**
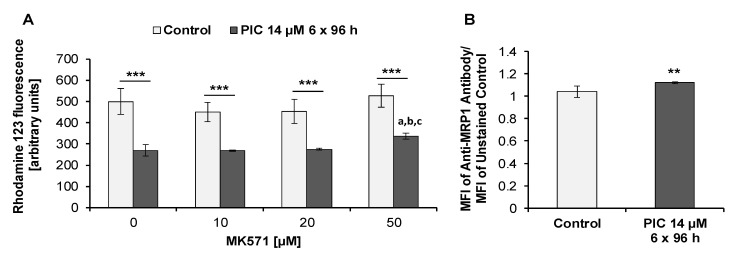
Detection of MRP1 in HL-60 cells. Cells were exposed to piceatannol (PIC, IC_90_ = 14 µM) for six repeated treatment cycles (6 × 96 h). (**A**) MRP1 activity analysis (flow cytometry analysis). Rhodamine 123 uptake/retention assay was performed in the presence and absence of MK571. Data are presented as means ± SD of three independent experiments in duplicates. *** *p* < 0.001, statistically significant differences between piceatannol-treated cells and untreated cells incubated in the absence or presence of MK571. ^a^ *p* < 0.001, statistically significant differences compared to piceatannol-treated cells incubated without MK571; ^b^ *p* < 0.001, statistically significant differences compared to piceatannol-treated cells incubated with 10 µM MK571; ^c^ *p* < 0.001, statistically significant differences compared to piceatannol-treated cells incubated with 20 µM MK571 (One-way ANOVA, Tukey’s post hoc analysis). (**B**) Expression of MRP1 in HL-60 cells. Control (untreated cells) and piceatannol-treated cells were stained with anti-MRP1 (QCRL-3) antibody (flow cytometry analysis). Data are presented as means ± SD of three independent experiments in duplicates. ** *p* < 0.01, statistically significant differences between piceatannol-treated cells and untreated cells (Mann–Whitney U-test).

In order to find out whether prolonged treatment with piceatannol enhances the effects on the activity and expression of MRP1, we decided to study these effects after six repeated treatment cycles, each lasting 96 h (Figure 9A). We found that under these conditions the intracellular Rho 123 level in piceatannol-treated cells decreased by about two-fold compared to control (untreated) cells. Thus, after six repeated treatment cycles with piceatannol Rho 123 efflux increased in HL-60 cells in comparison to 96 h exposure (Figure 8A and Figure 9A). In piceatannol-treated cells incubated in the presence of 50 µM MK571 a statistically significant increase in the intracellular Rho 123 level was noted compared to piceatannol-treated cells incubated in the presence of 10 µM and 20 µM MK571 or in the absence of MK571 (Figure 9A). Moreover, there were no statistically significant changes between control cells incubated in the absence of MK571 and control cells incubated in the presence of 10 µM, 20 µM, and 50 µM MK571 (Figure 9A).

After staining with the specific monoclonal anti-MRP1 antibody (clone QCRL-3) there was a statistically significant increase in the expression of MRP1 in piceatannol-treated cells compared to control cells (Figure 9B). The MFI shift for control cells was 1.04 (±0.05), whereas after exposure of cells to piceatannol (IC90) for six treatment cycles of 96 h each the MFI shift was 1.12 (±0.01). Thus, in comparison to 96 h exposure to piceatannol, after six repeated treatment cycles, MRP1 expression in HL-60 cells did not change (Figure 8B and Figure 9B).

### 2.7. Effects of Piceatannol on the Activity and Expression of BCRP

**Figure 10:**
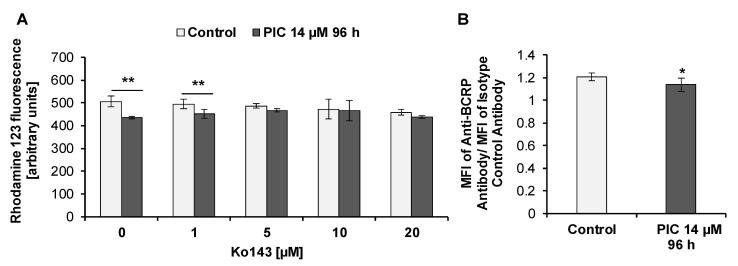
Detection of BCRP in HL-60 cells. Cells were incubated with piceatannol (PIC, IC_90_ = 14 µM) for 96 h. (**A)** BCRP activity analysis (flow cytometry analysis). Rhodamine 123 uptake/retention assay was performed in the presence and absence of Ko143. Data are presented as means ± SD of three independent experiments in duplicates. ** *p* < 0.01, statistically significant differences between piceatannol-treated cells and untreated cells, incubated in the absence or presence of Ko143 (One-way ANOVA, Dunnett’s post hoc analysis). (**B)** Expression of BCRP in HL-60 cells. Control (untreated cells) and piceatannol-treated cells were stained with anti-BCRP (clone 5D3) antibody or isotype control antibody (flow cytometry analysis). Data are presented as means ± SD of three independent experiments in duplicates. * *p* < 0.05, statistically significant differences compared to control (Student’s *t*-test).

We have previously found that Rho 123 efflux in piceatannol-treated MOLT-4 cells may be mediated by the BCRP protein [59]. Rho 123 is not a substrate for the wild-type variant of BCRP [76], however, it can be transported by mutant variants, e.g., BCRP isoforms resulting from mutations changing arginine at amino acid 482 to threonine (BCRP^R482T^) or glycine (BCRP^R482G^) [76]. We examined whether BCRP is able to mediate Rho 123 efflux in HL-60 cells. Rho 123 retention was assessed in the presence and absence of Ko143, a BCRP inhibitor [77]. The intracellular Rho 123 level in piceatannol-treated cells incubated with 1 µM, 5 µM, and 10 µM Ko143 slightly increased compared to piceatannol-treated cells incubated without Ko143 (Figure 10A). However, these changes were not statistically significant. Statistically significant differences were only observed between piceatannol-treated cells and untreated cells both incubated in the absence of Ko143 as well as between piceatannol-treated cells and untreated cells both incubated in the presence of 1 µM Ko143. In the presence of 5 µM, 10 µM, and 20 µM Ko143 Rho 123 retention was similar in both untreated and piceatannol-treated cells.

By using a specific monoclonal anti-BCRP antibody (clone 5D3) we detected the cell surface expression of BCRP in both control (untreated) and piceatannol-treated HL-60 cells (Figure 10B). We used the 5D3 monoclonal antibody that has the ability to recognize the wild-type BCRP as well as its mutated forms BCRP^R482T^ and BCRP^R482G^ [78,79]. MFI shift in control (untreated) cells was 1.21 (±0.03), whereas after 96 h of treatment of cells with piceatannol (IC_90_) MFI shift significantly decreased to 1.14 (±0.06) (Figure 10B).

## 3. Discussion

AML is a highly heterogeneous disease. Despite advances in the treatment of AML, overall long-term survival remains poor, especially for elderly patients [3,4]. New chemotherapeutic agents are needed to selectively target leukemic myeloid cells without major toxicity against non-malignant cells. Recently, polyphenolic compounds such as piceatannol have been reported to act as effective apoptosis inducers in different leukemia cell lines and thus appear to be promising chemotherapeutic agents in the treatment of leukemia [45,47,48,49,59]. The present study demonstrates that piceatannol is an apoptosis inducer and a multidrug resistance modulator in HL-60 human acute myeloid leukemia cells.

Our results revealed that piceatannol reduced the viability of HL-60 cells. We found that piceatannol at the IC_90_ concentration of 14 µM did not induce the cell cycle arrest in HL-60 cells; however, it increased the accumulation of cells in the sub-G1 fraction. In line with these findings, we have previously found that treatment of MOLT-4 human acute lymphoblastic leukemia cells with piceatannol at the IC_90_ concentration of 45.5 µM did not result in the cell cycle arrest but in an increase in the number of cells in the sub-G1 fraction [59]. Noteworthy, the IC_90_ concentration for HL-60 cells was much lower than for MOLT-4 cells. Thus, HL-60 acute myeloid leukemia cells appear to be more sensitive to piceatannol than MOLT-4 acute lymphoblastic leukemia cells. Similarly, other authors reported accumulation of cells in the sub-G1 phase after exposure of U937 human acute myeloid leukemia cells to piceatannol at the concentration of 10 µM, 20 µM, 40 µM, and 60 µM [48]. Moreover, in K562 human chronic myeloid leukemia cells 100 µM piceatannol induced the S-phase cell cycle arrest [49]. Interestingly, in non-leukemic cell lines, piceatannol led to the S-phase cell cycle arrest in Caco-2 human colon cancer cells and the G2/M-phase arrest in SK-Mel-28 human melanoma cells, whereas treatment of DU145 human prostate cancer cells and T24 and HT1376 human bladder cancer cells with this polyphenol resulted in the G0/G1-phase arrest [50,51,52,57].

Our results revealed that 14 µM piceatannol (IC_90_) did not induce oxidative stress in HL-60 cells, but rather decreased intracellular ROS generation. This finding confirmed our previous observation that 45.5 µM piceatannol (IC_90_) decreased intracellular ROS production in MOLT-4 cells [59]. Similar results concerning decreased ROS generation were also reported by other authors after treatment of HL-60 human leukemia cells and HepG2 human hepatoma cells with 50 µM, 100 µM, and 200 µM piceatannol [54]. Likewise, piceatannol at the concentration of 5 µM, 10 µM, and 50 µM decreased ROS generation in B16 murine melanoma cells [80]. Thus, mechanisms of piceatannol activity probably do not involve oxidative stress. Interestingly, other polyphenols elicited similar effects. For example, our previous studies showed that pterostilbene and resveratrol did not induce oxidative stress in HL-60 or MOLT-4 cells [81,82,83].

Autophagy has been shown to suppress tumor development [84,85]. On the other hand, under cellular stress conditions, e.g., nutrient deprivation, hypoxia, or treatment with anticancer drugs, autophagy can promote survival of cancer cells and resistance to chemotherapy [86,87,88]. Noteworthy, many polyphenols including piceatannol, resveratrol or pterostilbene have been reported to modulate autophagy in different cancer cell lines [59,60,82,83,89,90]. Our results showed that 14 µM piceatannol (IC_90_) did not induce autophagy in HL-60 cells since the LC3-II/LC3-I ratio indicated no conversion of LC3-I to LC3-II protein. In line with these results, immunofluorescence analysis did not show accumulation of autophagic vacuoles in piceatannol-treated HL-60 cells. In contrast to these findings, our previous report demonstrated that 45.5 µM piceatannol (IC_90_) induced autophagy in MOLT-4 cells. Other authors reported induction of autophagy by 30 µM piceatannol in U2OS human osteosarcoma cells [60].

We found that piceatannol induced apoptosis in HL-60 cells. Death of piceatannol-treated cells was accompanied by phosphatidylserine externalization, disruption of mitochondrial membrane potential, caspase-3 activation, internucleosomal DNA fragmentation, PARP1 cleavage, chromatin condensation, and fragmentation of cell nuclei. Noteworthy, piceatannol appeared to be an efficient apoptosis inducer in many leukemia cell lines including MOLT-4, U937, and THP-1 human leukemia cell lines [47,48,53,59]. Likewise, induction of apoptosis by piceatannol was demonstrated for other types of cancer cell lines such as DU145 human prostate cancer, HCT116, and HT29 human colorectal cancer as well as T24 and HT1376 human bladder cancer cell lines [46,50,58]. Thus, the in vitro studies suggest that piceatannol may be effective in eliminating cancer cells of a different type.

Acute myeloid leukemia cells have been demonstrated to acquire resistance to many chemotherapeutic agents, e.g., anthracyclines, cytarabine, or HMA [8,91]. Of note, HL-60 cells have been reported to acquire resistance to several drugs used in AML chemotherapy including cytarabine, anthracyclines doxorubicin, and daunorubicin as well as arsenic trioxide via mechanisms related to modulation of P-gp, MRP1, or BCRP expression [92,93,94]. Our results revealed that toxic effects exerted by piceatannol in HL-60 cells diminished after longer treatment periods, suggesting that the cells may acquire resistance to toxicity of this compound. The inhibition of rhodamine 123 efflux in piceatannol-treated HL-60 cells by MK571 suggested the involvement of ABC transporters belonging to the MRP family of proteins. This hypothesis was supported by the detection of MRP1 protein in piceatannol-treated HL-60 cells. Moreover, after six consecutive treatment cycles (6 × 96 h), Rho 123 efflux in piceatannol-treated HL-60 cells was enhanced in comparison to 96 h piceatannol exposure, suggesting that the activity of MRP1 increased after prolonged incubation with the compound. Rho 123 retention in piceatannol-treated HL-60 cells was not enhanced by CsA and Ko143, indicating that the ability of HL-60 cells to efflux Rho 123 did not result from the activity of P-gp or mutated BCRP (BCRP^R482T^ or BCRP^R482G^). In line with the aforementioned results, we did not detect the expression of P-gp in piceatannol-treated cells. However, we detected cell surface expression of BCRP, which was less pronounced after piceatannol exposure than in control cells. Noteworthy, Rho 123 is a substrate for mutant variants of BCRP, e.g., BCRP^R482T^ or BCRP^R482G^ [76]. Thus, the Rho 123 uptake/retention assay probably does not completely detect the activity of BCRP protein. On the other hand, the 5D3 monoclonal antibody we used in our experiments has the ability to recognize the wild-type BCRP as well as its mutated forms BCRP^R482T^ and BCRP^R482G^ [78,79]. We therefore detected BCRP expression, even though Rho 123 retention in piceatannol-treated cells increased only slightly in the presence of Ko143 compared to piceatannol-treated cells incubated without the inhibitor. Taking together the above findings, piceatannol targeted BCRP-positive HL-60 cells, whereas MRP-1-positive cells were probably resistant to piceatannol and were able to survive and reconstitute the cell population. Interestingly, BCRP is expressed in primitive leukemic hematopoietic cells which are considered to be leukemic stem cells involved in self-renewal of cell population [95]. This phenomenon has been suggested to contribute to intrinsic chemoresistance of these cells. Noteworthy, our previous report demonstrated that MOLT-4 cells acquired resistance to piceatannol toxicity [59]. Piceatannol targeted BCRP-positive MOLT-4 cells, whereas P-gp-positive MOLT-4 cells were probably resistant to piceatannol toxicity. Thus, MOLT-4 cells acquired resistance to piceatannol toxicity via different mechanisms than HL-60 cells. It is important to note that piceatannol appeared to be able to effectively eliminate BCRP-positive leukemia cells of two types, i.e., MOLT-4 human lymphoblastic leukemia cells [59] and HL-60 human myeloid leukemia cells. However, it seems to be ineffective in eliminating P-gp-positive or MRP1-positive subpopulations of leukemia cells. One possible explanation may be that piceatannol is a substrate of MRP1 protein and P-gp. Interestingly, piceatannol has been suggested to act as MRP1 inhibitor since it inhibited efflux of BCECF, a substrate for MRP1, from human erythrocytes [96]. However, the mechanism of inhibition is unknown. Thus, it cannot be excluded that piceatannol is a competitive MRP1 inhibitor and under some conditions may act as MRP1 substrate.

In summary, our results indicate that piceatannol induces caspase-dependent apoptosis in HL-60 human myeloid leukemia cells and appears to be a promising chemotherapeutic agent in the treatment of AML. Moreover, to our knowledge, this is the first study demonstrating that HL-60 cells are able to acquire resistance to piceatannol toxicity and the activity of the MRP1 protein may be related to this phenomenon. However, in vivo studies with animal models are needed to corroborate the hypothesis that leukemic cells are able to acquire resistance to piceatannol toxicity. Our results highlight the importance of research into the effects of polyphenols on ABC transporters activity and expression, since the new data provided in this study indicate that the use of piceatannol as a potential chemotherapeutic agent may be associated with the risk of MDR. However, it is important to note that MDR is a widely observed phenomenon that accompanies treatment of different cancer cells with a variety of drugs. Cancer cells tend to activate resistance mechanisms in order to survive [86,87,88]. Thus, each cytotoxic agent is a potential substrate for ABC transporters and treatment of cancer cells with piceatannol as well as with other cytotoxic agents is associated with the risk of MDR. Successful chemotherapy for AML or other malignancies may require a combination of chemotherapeutics cytotoxic against subpopulations of cells expressing different ABC transporters. Our findings imply that piceatannol is able to eliminate BCRP-positive HL-60 myeloid leukemia cells, whereas MRP1-positive cells are probably resistant to piceatannol toxicity. However, piceatannol may be used in combination with other chemotherapeutic agents to eliminate MRP1-positive leukemia cells. Additionally, piceatannol can be used in experimental models to study molecular pathways involved in multidrug resistance of cancer cells.

In conclusion, piceatannol induces caspase-dependent apoptosis in HL-60 human myeloid leukemia cells, however, HL-60 cells are able to acquire resistance to piceatannol toxicity via mechanisms related to MRP1 activity. Piceatannol appears to be a promising chemotherapeutic agent in the treatment of AML. Its use in combination with other chemotherapeutic agents may help overcome the risk of multidrug resistance.

## 4. Materials and Methods

### 4.1. Chemicals

Piceatannol was obtained from Sigma-Aldrich (St. Louis, MO, USA). Piceatannol stock solutions were prepared in dimethyl sulfoxide (DMSO) and diluted to indicated concentrations before use. Rhodamine 123 and Hoechst 33342 were purchased from Thermo Fisher Scientific (Waltham, MA, USA). Propidium iodide was from Sigma-Aldrich (USA) and BD Pharmingen™ (San Diego, CA, USA). 7AAD (7-aminoactinomycin D) was from BD Pharmingen™ (USA). JC-1 (5,5′,6,6′-tetrachloro-1,1,3,3′-tetraethylbenzimidazolylcarbocyanine iodide) was purchased from Calbiochem (Burlington, MA, USA). Neutral red and cyclosporin A were obtained from Sigma-Aldrich (USA). Ko143 and MK571 were from Cayman Chemical (Ann Arbor, MI, USA). H_2_DCFDA (2′,7′-dichlorodihydrofluorescein diacetate) was obtained from Molecular Probes (Eugene, OR, USA). Rabbit anti-LC3 antibody (cat. no. PM036) was from Mcedical & Biological Laboratories Co. (MBL, Nagoya, Japan). Rabbit anti-PARP1 antibody (cat. no 9542) was obtained from Cell Signaling Technology (Danvers, MA, USA). Cy3-conjugated goat anti-rabbit antibody was purchased from Jackson ImmunoResearch Laboratories, Inc. (West Grove, PA, USA). Horseradish peroxidase-conjugated mouse anti-GAPDH antibody, horseradish peroxidase-conjugated goat anti-rabbit antibody were purchased from Sigma-Aldrich (USA). PE-conjugated mouse monoclonal IgG_2a_ CD243 (ABCB1) antibody (clone UIC2; cat. no A18376), isotype control—PE-conjugated normal mouse IgG_2a_ antibody (cat. no MA1-10425) were obtained from Thermo Fisher Scientific. FITC-conjugated mouse monoclonal IgG_2a_ anti-MRP1 antibody (QCRL-3; cat. no 557593) and isotype control—FITC-conjugated mouse IgG_2a_ antibody (cat. no 555573) were from BD Pharmingen™ (USA). PE-conjugated mouse monoclonal IgG_2b_ ABCG2 antibody (5D3; cat. no sc-18841), isotype control—PE-conjugated normal mouse IgG_2b_ antibody (cat. no sc-2868) were from Santa Cruz Biotechnology, Inc. (Dallas, TX, USA). All other reagents, obtained from commercial suppliers, were of analytical grade.

### 4.2. Cell Culture

The HL-60 human promyelocytic leukemia cell line was obtained from the American Type Culture Collection (Rockville, MD, USA). HL-60 cells were maintained at 37 °C, in a humidified atmosphere containing 5% CO_2_. Cells were cultured in RPMI 1640 medium (Sigma-Aldrich, USA) supplemented with 10% heat-inactivated fetal bovine serum (FBS, Sigma-Aldrich, USA), 2 mM L-glutamine, 100 IU/mL penicillin (Sigma-Aldrich, USA), and 100 μg/mL streptomycin (Sigma-Aldrich, USA).

### 4.3. Cytotoxicity Assay

The cytotoxic effect of piceatannol on HL-60 cells was examined after 72 h of treatment with this compound using the neutral red uptake assay, as described previously [82,83]. The concentration of piceatannol required to inhibit the growth of HL-60 cells by 50% (IC_50_) and 90% (IC_90_) was calculated based on the dose-response curve.

### 4.4. Cell Cycle Analysis

HL-60 cells were treated with piceatannol (IC_90_ = 14 µM) for 6, 12, 24, 48, and 72 h. Cells not exposed to piceatannol were used as control. After treatment, cells (2 × 10^6^ cells per sample) were collected, washed with cold phosphate-buffered saline (PBS), fixed in ice-cold 70% ethanol, stained with propidium iodide (PI), and analyzed by flow cytometry (Becton Dickinson FACScan, Franklin Lakes, NJ, USA), as described previously [59,82,83]

### 4.5. Annexin V-FITC/PI Assay

To examine phosphatidylserine externalization and loss of plasma membrane integrity in piceatannol-treated HL-60 cells Annexin V-FITC Apoptosis Detection Kit (BD Pharmingen™, USA) was used, as described previously [82,83].Cells were treated with piceatannol (IC_90_ = 14 µM) for 12, 24, 48, 72, and 96 h. Cells not exposed to piceatannol were used as control. After treatment, cells (5 × 10^5^ cells per sample) were collected and stained with FITC-conjugated annexin V and PI according to the manufacturer’s protocol. Next, samples were analyzed by flow cytometry (Becton Dickinson FACScan, USA).

### 4.6. Caspase-3 Activity Assay

To measure caspase-3 activity in piceatannol-treated HL-60 cells, FITC-conjugated Monoclonal Active Caspase-3 Antibody Apoptosis Kit I (BD Pharmingen™, USA) was used, as described previously [82,83] Cells were exposed to piceatannol (IC_90_ = 14 µM) for 48 h. Cells not exposed to piceatannol were used as control. Following treatment, cells (5 × 10^5^ cells per sample) were collected and stained with FITC-conjugated anti-active caspase-3 antibody according to the manufacturer’s protocol, and samples were analyzed by flow cytometry (Becton Dickinson FACScan, USA).

### 4.7. Analysis of the Mitochondrial Membrane Potential

Analysis of the mitochondrial membrane potential in piceatannol-treated HL-60 cells was performed using JC-1 dye, as described previously [82,83,97]. HL-60 cells were exposed to piceatannol (IC_90_ = 14 µM) for 48 h. Cells not exposed to piceatannol were used as control. After treatment, cells (5 × 10^5^ cells per sample) were collected and stained with JC-1. Samples were analyzed by flow cytometry (Becton Dickinson FACScan, USA).

### 4.8. DNA Fragmentation Assay

DNA fragmentation in piceatannol-treated HL-60 cells was determined by agarose gel electrophoresis. Cells were treated with piceatannol (IC_90_ = 14 µM) for 12, 24, 48, and 72 h. Cells not exposed to piceatannol were used as control. Following treatment, cells (2 × 10^6^ cells per sample) were collected and DNA isolation was performed, as described previously [82,83,97]. DNA fragments were fractionated by electrophoresis in 1.8% agarose gel, stained with ethidium bromide, and examined using Gel Doc2000 (Bio-Rad, Milan, Italy).

### 4.9. Detection of Intracellular Production of Reactive Oxygen Species

Detection of ROS production in piceatannol-treated HL-60 cells was performed using H_2_DCFDA (final concentration 10 µM), as described previously [59,82,83]. HL-60 cells (5 × 10^5^ cells per sample) were exposed to piceatannol (IC_90_ = 14 µM) for 45 min, 2 h, 4 h, and 6 h. Cells not exposed to piceatannol were used as control. Samples were analyzed for DCF fluorescence by flow cytometry (Becton Dickinson FACSCalibur, USA).

### 4.10. Western Blotting Analysis

HL-60 cells were treated with piceatannol (IC_90_ = 14 µM) for 24, 72, and 96 h. Cells not exposed to piceatannol were used as control. Western blotting was performed as described previously [59,82,83]. Protein samples were separated electrophoretically by SDS-PAGE (12% for LC3, 10% for PARP1) and transferred onto PVDF membrane. The following antibodies were used: rabbit anti-LC3 primary antibody (1:4000), rabbit anti-PARP1 primary antibody (1:1000), horseradish peroxidase-conjugated anti-rabbit secondary antibodies (1:10,000), and horseradish peroxidase-conjugated anti-GAPDH antibody (1:50,000). The enhanced chemiluminescence method using the Chemiluminescent Peroxidase Substrate (Sigma-Aldrich, USA) was used to detect the bound antibodies. The Quantity One Software 4.6.6 (Bio-Rad, Hercules, CA, USA) was used to perform the densitometric analysis of immunoreactive protein bands.

### 4.11. Immunofluorescent Analysis

The immunofluorescent analysis of piceatannol-treated HL-60 cells was performed as described previously [59,82,83]. Cells were exposed to piceatannol (IC_90_ = 14 µM) for 24, 48, and 72 h. Cells not exposed to piceatannol were used as control. Equal numbers (10^5^ cells) of control cells and piceatannol-treated cells were cytocentrifuged onto microscopic poly-L-lysine coated slides (Sigma-Aldrich, USA). Cells were then incubated with rabbit anti-LC3 primary antibody (1:500) and subsequently with Cy3-conjugated anti-rabbit secondary antibody (1:600). Afterwards, cells were stained with Hoechst 33342 (final concentration 1 µg/mL) and examined by a fluorescence microscope (Nikon Eclipse E800, Nikon Corp., Tokyo, Japan) using × 60 oil immersion objective lens.

### 4.12. Rhodamine 123 Uptake/Retention Assay

Rhodamine 123 uptake/retention assay was performed as described previously [59], with some modifications. HL-60 cells were exposed to piceatannol (IC_90_ = 14 µM) for 96 h or six consecutive treatment cycles each lasting 96 h (6 × 96 h). Cells not exposed to piceatannol were used as control. After exposure, cells (5 × 10^5^ cells per sample) were collected, centrifuged, and supernatants were discarded. Following washing with RPMI 1640 medium, cells were suspended in RPMI 1640 medium containing 0.1 µg/mL rhodamine 123 and incubated for 1 h at 37 °C in the dark in the presence or absence of an appropriate ABC-transporter inhibitor. The following inhibitors of ABC-transporters were used: cyclosporin A, MK571, or Ko143. Cyclosporin A at the concentration of 2, 5, and 10 µM was used to examine P-gp activity. MK571 at the concentration of 10, 20, and 50 µM was used for MRP1 activity analysis. Ko143 at the concentration of 1, 5, 10, and 20 µM was used to study BCRP activity. After incubation, cells were washed twice with ice-cold rhodamine 123-free culture medium with or without an appropriate inhibitor. Afterwards, cells were incubated in rhodamine 123-free medium for 45 min at 37 °C, in the presence or absence of an appropriate ABC transporter inhibitor. Following centrifugation (300 *g*/5 min/4 °C) supernatants were discarded, cells were suspended in the culture medium containing 0.25 µg/mL 7AAD and incubated in the presence or absence of an appropriate inhibitor for 10 min at RT in the dark. After incubation, samples were kept on ice and immediately analyzed by flow cytometry (Becton Dickinson FACSCalibur, USA). 7AAD-positive cells, corresponding to non-viable cells, were excluded from data analysis. RPMI 1640 medium used in the experiment did not contain phenol red and was supplemented with 10% FBS, 2 mM L-glutamine, and antibiotics. Experiments based on the exposure of HL-60 cells to six consecutive treatment cycles each lasting 96 h (6 × 96 h) were performed in the following way. Cells were treated with piceatannol (IC_90_). After each 96-h treatment period, cells were collected, washed, re-planted into a new flask and re-exposed to piceatannol for a further 96 h. In other words, cells were treated with piceatannol (IC_90_) for 24 days and after each 96 h period the medium was replaced with fresh medium containing piceatannol (IC_90_). Following all treatment cycles, rhodamine 123 uptake/retention assay was performed.

### 4.13. Detection of P-gp

Detection of P-gp in HL-60 cells was performed as described previously [59], with some modifications. HL-60 cells were exposed to piceatannol (IC_90_ = 14 µM) for 96 h. Cells not exposed to piceatannol were used as control. After exposure, cells (5 × 10^5^ cells per sample) were collected and washed with cold Stain Buffer (BD Pharmingen™, USA). HL60 cells have been reported to express Fc receptors [98]. In order to prevent potential nonspecific binding of monoclonal antibodies to Fc receptors and eliminate erroneous results in flow cytometry [99], Fc Block (BD) was used to block these receptors. Following incubation with Fc Block for 10 min at room temperature (RT), cells were incubated on ice for 30 min in the dark with PE-conjugated mouse monoclonal antibody against P-gp (UIC2) or PE-conjugated mouse IgG_2a_ isotype control antibody. The final concentration of the UIC2 antibody was 5 µg/mL (0.5 µg per 5 × 10^5^ cells in 100 µL) and was equal to the final concentration of the isotype control antibody. After incubation, cells were washed with cold Stain Buffer and incubated with 0.5 µg/mL 7AAD for 10 min at RT in the dark. Next, samples were immediately analyzed by flow cytometry (Becton Dickinson FACSCalibur, USA). 7AAD-positive cells, corresponding to non-viable cells, were excluded from data analysis. Results were expressed as the ratio of the mean fluorescence intensity (MFI) of the UIC2 antibody and the isotype control antibody (MFI shift).

### 4.14. Detection of MRP1

Detection of MRP1 in HL-60 cells was performed as described previously [59], with some modifications. HL-60 cells were exposed to piceatannol (IC_90_ = 14 µM) for 96 h or for 6 consecutive treatment cycles each lasting 96 h (6 × 96 h). Cells not exposed to piceatannol were used as control. After treatment, cells (5 × 10^5^ cells per sample) were collected and washed with cold PBS. To block Fc receptors and minimize the background fluorescence resulting from nonspecific antibody binding, cells were incubated with 10% human AB serum for 20 min at 4 °C. Next, cells were washed with cold PBS, suspended in Cytofix/Cytoperm^TM^ solution (BD Biosciences, Franklin Lakes, NJ, USA), and incubated on ice for 20 min. After washing with Perm/Wash^TM^ Buffer (BD Biosciences, USA), cells were incubated for 30 min at 4 °C in the dark with FITC-conjugated mouse monoclonal antibody against MRP1 (IgG_2a_, clone QCRL-3) or FITC-conjugated mouse IgG_2a_ isotype control antibody. Since the FITC-conjugated mouse monoclonal anti-MRP1 antibody (QCRL-3) reacts with an intracellular epitope of MRP1, the procedure for intracellular staining was used. The anti-MRP1 antibody was recommended by the manufacturer to be used in flow cytometric analysis together with the FITC-conjugated mouse IgG_2a_ isotype control antibody. The final concentration of the QCRL-3 antibody was 3 µg/mL (0.3 µg per 5 × 10^5^ cells in 100 µL) and was equal to the final concentration of the isotype control antibody. Following washing with Perm Wash Buffer, samples were immediately analyzed by flow cytometry (Becton Dickinson FACSCalibur, USA). The FITC-conjugated mouse IgG_2a_ isotype antibody appeared to have high nonspecific binding in our experiments. Cells stained with this antibody had higher fluorescence intensity than unstained cells or cells stained with the anti-MRP1 (QCRL-3) antibody. Therefore, results were expressed as the ratio of MFI of the QCRL-3 antibody and the unstained control (MFI shift). Isotype controls have been widely used in flow cytometry assays. However, there are reports suggesting that sometimes such controls may be unreliable [99,100].

### 4.15. Detection of BCRP

Detection of BCRP in HL-60 cells was performed as described previously [59], with some modifications. HL-60 cells were exposed to piceatannol (IC_90_ = 14 µM) for 96 h. Cells not exposed to piceatannol were used as control. Following treatment, cells (5 × 10^5^ cells per sample) were collected and washed with cold PBS. To block Fc receptors, cells were incubated with 10% human AB serum for 20 min at 4 °C. Next, cells were washed with cold PBS and incubated on ice for 30 min in the dark with PE-conjugated mouse monoclonal antibody against BCRP (5D3) or PE-conjugated mouse IgG_2b_ isotype control antibody. The final concentration of the 5D3 antibody was 5 µg/mL (0.5 µg per 5 × 10^5^ cells in 100 µL) and was equal to the final concentration of the isotype control antibody. After incubation, cells were washed with cold PBS and incubated with 0.5 µg/mL 7AAD for 10 min at RT in the dark. Afterwards, samples were immediately analyzed by flow cytometry (Becton Dickinson FACSCalibur, USA). 7AAD-positive cells, corresponding to non-viable cells, were excluded from data analysis. Results were expressed as the ratio of MFI of the 5D3 antibody and the isotype control antibody (MFI shift).

### 4.16. Statistical Analysis

Statistical analysis was performed using Statistica 13 software (StatSoft Polska, Kraków, Poland). Data are expressed as means ± SD. Statistical differences between samples were evaluated using the Mann–Whitney U test, Student’s *t*-test, One-way ANOVA and Tukey’s or Dunnett’s post hoc tests. Differences were considered significant at *p* < 0.05, *p* < 0.01, *p* < 0.001, and *p* < 0.0001.

## Data Availability

Not applicable.
